# Susceptibility to Insecticides and Natural Infection in Aedes aegypti: An Initiative to Improve the Mosquito Control Actions in Boyacá, Colombia

**DOI:** 10.5334/aogh.2805

**Published:** 2020-08-06

**Authors:** Omar Cantillo-Barraza, Manuel Medina, Yurany Granada, Camilo Muñoz, Cesar Valverde, Fernando Cely, Paola Gonzalez, Yovanny Mendoza, Sara Zuluaga, Omar Triana-Chávez

**Affiliations:** 1 Grupo Biología y Control de Enfermedades Infecciosas, BCEI, Universidad de Antioquia, Medellín, CO; 2 Programa de control de enfermedades transmitidas por vectores, Secretaria de Salud Departamental, Tunja, Boyacá, CO

## Abstract

**Background:**

Integrated management strategies for dengue prevention and control have been the main way to decrease the transmission of arboviruses transmitted by *A. aegypti* in Colombia. However, the increase of chikungunya (CHIKV), Zika, and dengue (DENV) fever cases suggests deficiencies in vector control strategies in some regions from this country.

**Objective:**

This work aimed to establish a baseline susceptibility profile of *A. aegypti* to insecticides, determine the presence of *kdr* mutations associated with resistance to pyrethroids, and detect natural arbovirus infection in this vector from Moniquirá – Boyacá, one of the most endemic cities in Colombia.

**Methods:**

Mosquitos were collected in six neighborhoods, and colonies established in the laboratory. Susceptibility to malathion and lambda-cyhalothrin insecticides was evaluated, and we examined the point mutations present in portions of domains I, II, III, and IV of the sodium channel gene using a simple allele-specific PCR-based assay (AS-PCR).

**Findings:**

*A. aegypti* from Moniquirá showed decreased susceptibility to pyrethroid insecticides, and kdr mutations 419L, 1016I, and 1558C with allelic frequencies of 0.39, 0.40 and 0.95, respectively, were observed. The minimal infection rate (MIR) to DENV-1 was 44.1, while to CHIKV was 14.7.

**Conclusions:**

We establish a baseline insecticide resistance, *kdr* mutations, and arbovirus circulation, which contain the elements necessary for the consolidation of a local surveillance strategy with an early warning system and rational selection and rotation of insecticides.

## Introduction

*Aedes* (Stegomyia) *aegypti* is the primary vector of dengue, Zika, and chikungunya viruses. These arboviruses affect millions of people around the world but mostly in the tropical and subtropical zones from Asia, Africa, and America, where temperature and humidity favor the mosquito proliferation [1,2]. In the Andean region, Colombia is the country most affected by these diseases, due mainly to a wide distribution of *A. aegypti*, presence of social factors that generate unplanned urbanization, high human population movement, changes in the habits of domestic water use, and lack of public health policies to implement efficient control measures [3].

Dengue transmission in the Department of Boyacá is endemic-epidemic with the occurrence of frequent outbreaks [4]. During 2016 and 2017, this Colombian region reported a dengue incidence above the national average with 1,600 and 243.9 cases per 100,000 inhabitants, respectively [5,6]. The municipality of Moniquirá presented the highest incidence of dengue cases during this period, with the occurrence of autochthonous cases of chikungunya. Due to the epidemiological situation, this municipality has been prioritized by departmental public health agencies for the strengthening of intervention and surveillance actions to reduce the incidence of both arboviruses [6].

The usage of organophosphate and pyrethroids insecticides is the main activity used for the reduction of mosquito populations in epidemic periods. However, the indiscriminate use of these, the lack of monitoring of susceptibility in natural mosquitos’ populations, and the deficiency in rotation policies have led to the development of resistance in some parts of the country. In Colombia, the metabolic resistance, as well as the knockdown resistance (*kdr*) has been widely reported [7,8,9,10]. In the last years, the presence of three mutations in the gene coding for the sodium channel, which confer resistance to pyrethroid insecticides in Colombian *A. aegypti* natural populations, was described [7,10,11,12].

The implementation of alternatives in epidemiological surveillance such as the viral infection of the mosquito, the determination of the susceptibility status to insecticides, and the monitoring of *kdr* mutations could contribute to the improvement of local intervention programs. In this work, we described the use of these measures in the municipality of Moniquirá intending to strengthen the control and surveillance actions against this vector.

## Materials and methods

### Study area

**Figure 1:**
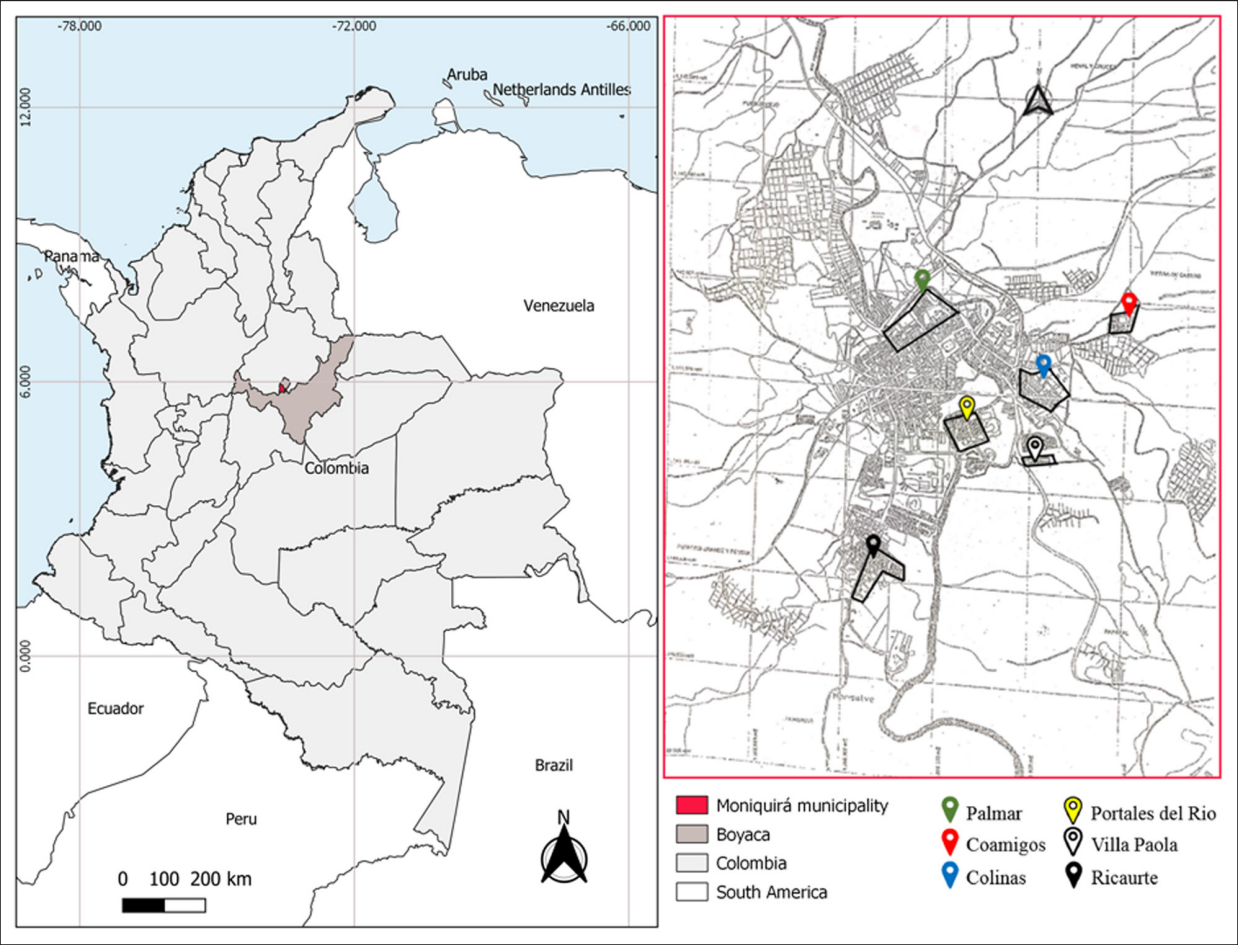
Geographic location of the municipality of Moniquirá (area highlighted in red on the main map), department of Boyacá.

The study was conducted during 2017 in six neighborhoods of the municipality of Moniquirá, Boyacá, Colombia (5°52’33.55”N, 73°34’24.92”W), at an altitude of 1700 m, precipitation of 2005 mm and an average temperature of 19°C (Figure 1). The collection of mosquitoes was limited to these neighborhoods according to the reports of the occurrence of clinical cases and the abundance of mosquitoes recorded by the Departmental Health Secretary during 2016.

### Mosquito collection

Both adult mosquitoes and immature stages were collected with the assistance of staff involved in vector-borne diseases programs from Health Secretary of Boyacá. In each dwelling visited, an active adult search was conducted for 15 minutes using sweep nets and oral aspirators in bedrooms, bathrooms, living rooms, and other places where the owners reported the presence of mosquitoes. The mosquitoes collected were transported to the Laboratory of Biology and Control of Infectious Diseases (BCEI) in transparent containers, which contained 10% sucrose in distilled water. Subsequently, the mosquitoes were identified at the species level using the taxonomic key of the Walter Reed Military Research Institute (WRAIR), developed by Rueda in 2004 [13]. Finally, pools of three to four individuals were stored at –80°C until molecular analysis was performed. The larvae and pupae were reared until adults emerged and maintained under controlled conditions of temperature (26 ± 1°C), relative humidity (80 ± 5%), and photoperiod (12 h light: 12 h dark).

### Insecticides bioessays

The biological assays were carried out following the methods proposed by the Center for Disease Control and Prevention (CDC) of Atlanta, the United States, and the World Health Organization, for adults and larvae, respectively [14,15]. Around 20 adult females of F1 filial generation were exposed to malathion with a purity of 98.7% (Fluka Analytical, St. Louis, USA), diluted in absolute ethanol (Merck) to a concentration of 50 ppm [15]. Briefly, the female mosquitoes were introduced in 250 ml Schott® bottles, previously impregnated with 1 ml of the insecticide. Mortality was monitored every five min and recorded at 30 and 60 min post-treatment [9,16]. As a control, bottles impregnated with ethanol were used. For the evaluation of susceptibility of larvae, the insecticide lambda-cyhalothrin with purity 97.8% was used (Fluka Analytical, St. Louis, USA), which was diluted in ethanol (100 mL) until obtaining a stock concentration at 100 ppm. From this concentration, different solutions were prepared that caused mortality between 2% and 99% of the larvae. For each bioassay, around 20 *A. aegypti* third- or fourth-instar F1 larvae, were exposed to six concentrations of the insecticide. Mortality was assessed at 24 h, and the results were analyzed based on WHO’s criteria for resistance to insecticides [17]. All bioassays were performed simultaneously with the insecticide susceptible Rockefeller strain, which was used as a reference. Each trial was performed in triplicate, with three biological repetitions each.

### DNA extraction and genotyping of kdr mutations

Adults mosquitoes collected in the field and those from larvae emerged in the laboratory were selected for genomic DNA extraction. The mosquitoes were individualized in 1.5 ml vials, and the total DNA was extracted using the ZR Tissue & Insect DNA MiniPrep kit (ZYMO RESEARCH, Irvine, CA, USA). To genotype the mutations V419L, V1016I, and F1558C of the sodium channel gene-coding region, an allele-specific PCR (AS-PCR) was used. For the V1016I and F1558C mutations, the primers reported by Chun-Xiao Li were used except for the primer that identifies the mutated allele for position 1016 reported by Granada et al., 2018 [10,18]. For the V419L mutation, the primers reported by Granada et al., 2018 were used. For each of the mutations, two PCRs were carried out, one of them with the primer that identified the wild-type allele and the other with the primer corresponding to the mutated allele. Each reaction was performed in a 25 μL volume consisting of 2 μL of DNA, 2.5 μL of KAPA buffer [10×], 1 μL of dNTPs [10 mM], 1 μL of MgCl_2_ [25 mM], 2 μL of each of the primers [10 mM], 0.2 μL of KAPA Taq polymerase and 14.3 μL of water. The amplification reactions were performed at initial denaturation step of 30 s at 94°C, followed by 35 cycles of 30 s at 94°C, 60 s at 60°C or 62°C (60°C for the mutation at position 419 and 1016, and 62°C for the mutation at position 1558), and 60 s at 72°C, with 7 min at 72°C for the final extension, according to the KAPA kit instructions (Kapa Biosystems, Boston, MA, USA). PCR amplification products were separated on a 2.5% agarose gel in a TE buffer, at 100 V for 60 minutes.

### Extraction and amplification of viral RNA

For the detection of arboviruses in mosquitoes, the above-described pools were processed as follows. RNA extraction was performed using the commercial kit RNeasy Mini Kit (Qiagen, Duesseldorf, Germany). For this, each pool was macerated mechanically following the manufacturer’s instructions (Qiagen®). The multiplex RT-PCR was performed in a single step with the Luna Universal One-Step RT-qPCR kit (Biolabs®). Each ten μL reaction contained one μL of RNA, 0.5 μL of enzyme mix at 1×, five μL of reaction mix at 1× and 0.1 μL of each of the primers at 0.4 μM, and 3.3 μL of water grade molecular biology. To diagnose dengue virus, the primers forward DV3-5’-AARTGIGCYTCRTCCAT-3’ and reverse DSP1-5’-AGTTTCTTTTCCTAAACACCTCG-3’, DSP2-5’-CCGGTGTGCTCRGCYCTGAT-3’, DSP3-5’-TTAGAGTYCTTAAGCGTCTCTTG-3’, and DSP4-5’-CCTGGTTGATGACAAAAGTCTTG-3’, which amplify fragments of 169, 362, 265, and 426 bp of the NS3 gene of the DENV-1, DENV-2, DENV-3 and DENV-4 serotypes, respectively, were used [19]. Additionally, a mixture of the primers ZIKF-5’-CCTTGGATTCTTGAACGAGGA-3’ and ZIKR-5’-AGAGCTTCATTCTCCAGATCAA-3’ were added, which amplify a region of 192 bp specific for the NS5 gene of Zika virus; and the primers CHIKVF-E1-5’-TACCCATTCATGTGGGGC-3’ and CHIKVR-E1-5’-GCCTTTGTACACCACGATT-3’ which amplifies a region of 294 bp specific for the E1 gene of chikungunya virus [20,21]. Positive samples were confirmed with a species-specific RT-PCR, using only the specific primers for each virus or serotype. For RT-PCR, the following thermal profile was used: reverse transcription 10 min at 55°C, with an initial denaturation 1 min at 95°C, followed by 40 cycles of 10 s at 95°C, 30 s at 56°C, and 20 s at 72°C. Negative controls for extraction and amplification were included in each reaction, and RNA from the dengue (each serotype), Zika, and chikungunya viruses from the supernatant of infected VERO cells were included as a positive control. The controls were processed simultaneously and under the same conditions as the samples. PCR amplification products were analyzed on a 2.5% agarose gel in a TBE buffer 0.5×. The minimum infection rate (MIR) for viruses detected in adults captured in the field was calculated as the number of positive pools/total number of mosquitoes processed * 1000 [22].

### Genotypic and allelic frequencies

The genotype for each mosquito was deduced from the electrophoretic profiles. With this data, the genotypic and allelic frequencies of each mutation were calculated. The observed and expected genotypic frequencies were compared through a Hardy-Weinberg equilibrium analysis using the GENEPOP software version 4.2, to evaluate possible selection pressures.

### Statistic analysis

The adult insecticides bioassay was carried out following the mortality criteria defined by the CDC methodology [16]. For this, the mortality was evaluated at a diagnostic time of 30 min in contact with the insecticide, in the following way: if the mortality was less than 80%, the population was considered as resistant; if mortality was equal to or greater than 98%, the population was susceptible; and if the mortality was between 80% and 97%, the population is cataloged with the possibility of resistance and should be kept under surveillance. The mosquitoes were monitored for an additional 30 min to confirm the mortality.

Regarding the larvae, the lethal dose 50 (LD_50_) and the values of larval mortality percentages obtained for each of the concentrations at 24 h, were subjected to a log regression analysis probit using the statistical package SPSS version 24 [23]. The resistance ratio (RR) was calculated by dividing the LD_50_ of the “Moniquirá” strain over the LD_50_ of the Rockefeller reference strain. The RR obtained was interpreted as susceptible (<5 times); moderate (between 5 to 10 times), and resistant (>10 times) [24].

## Results

### Presence of mosquitoes

All neighborhoods sampled were positive for the presence of the different stages of the biological cycle of *A. aegypti*. The number of adults obtained in the laboratory from larvae captured in the field was 530, of which 273 (51.5%) were females and 257 (48.5%) males. Of the 21 ovitraps installed in the area, 24 eggs were found, of which 19 hatched (79.2%). With this material, the “Moniquirá” strain was founded.

### Susceptibility to insecticides

**Figure 2:**
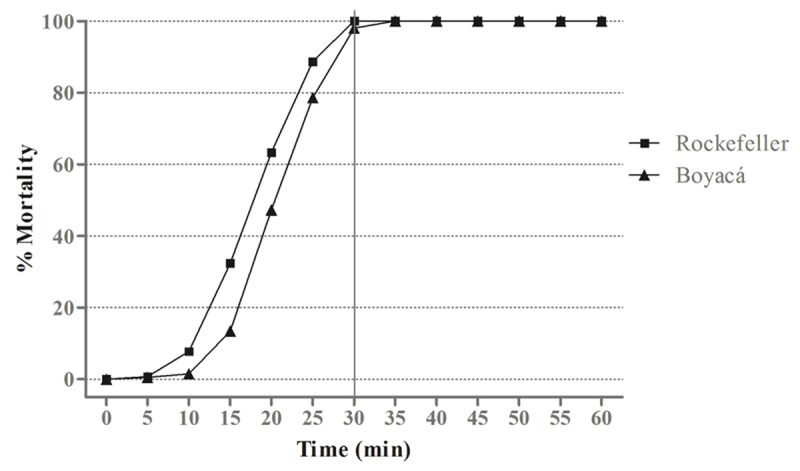
Susceptibility of adults of Moniquirá *A. aegypti* strain to malathion insecticide. On the left “y” axis the adult mortality for malathion is shown at a diagnostic dose and time of 50 μg/mL and 30 minutes, respectively.

The Moniquirá strain presented a malathion susceptibility profile with a mortality percentage of 98% at 30 min of diagnostic time (Figure 2). Likewise, it showed moderate resistance to the lambda-cyhalothrin insecticide with RR = 8.7. Table 1 summarizes the concentrations of the insecticide used, and the values of the LD_50_ and LD_90_ obtained.

**Table 1:** Resistance profile to lambda-cyhalothrin of *Aedes aegypti* mosquitoes from Moniquirá, Boyacá, Colombia.

Doses (ppm)	N° larvae	Death Larvae/total	Mortality (%)

0.00188	20	35/180	19.44
0.00375	20	73/180	40.55
0.00750	20	143/180	79.44
0.01500	20	168/180	93.33
0.03000	20	177/180	98.33
0.06000	20	179/180	99.44

LD_50_: 0,004124 Lim(0,002–0,006); LD_90_: 0,013 Lim(0,013–0,016).

### Mosquitoes from Moniquirá are carrying at least one kdr mutation in the sodium channel gene

Thirty-six mosquitoes of *A. aegypti* were analyzed for the presence of three *kdr* mutations in the sodium channel gene associated with insecticide resistance, which were previously reported in Colombia (V419L, V1016I, and F1558C). The three possible genotypes of the V419L (V/V, V/L, and L/L), and V1016I (V/V, V/I, and I/I) mutations were found in *A. aegypti* mosquitoes from Moniquirá (Table 2). The mutated allele frequency at 419 and 1016 positions was 0.39 and 0.4, respectively. By contrast, almost all the mosquitoes presented the mutated allele at the position 1558 (Table 2). Furthermore, the *A. aegypti* population from Moniquirá was found in Hardy-Weinberg equilibrium for the three loci evaluated (Table 2). Interestingly, 100% of the individuals assessed had at least one of the mutated alleles, and 24 of the individuals evaluated (70.5%) had at least two mutated alleles. Finally, 8.8% (3/34) showed a homozygous triple mutated genotype.

**Table 2:** Allele and genotype frequencies and associated P values for chi-squared tests for deviation from Hardy-Weinberg equilibrium of sodium channel mutations in *Aedes aegypti* from Moniquirá, Boyacá; Colombia.

Mutation	n	Allelic frequency		Genotype			Hardy-Weinberg equilibrium analysis			

		Wild	Mutated	Wild Homozygous	Heterozygous	Mutated homozygous	Ho	He	X^2^	P

419	36	0.61	0.39	0.33	0.56	0.11	0.56	0.48	0.0285	0.48
1016	34	0.60	0.40	0.32	0.56	0.12	0.56	0.48	0.0279	0.41
1558	36	0.06	0.94	0.00	0.11	0.89	0.11	0.10	0.0035	1.00

### Natural infection of A. aegypti shows infection with DENV-1 and CHK

**Figure 3:**
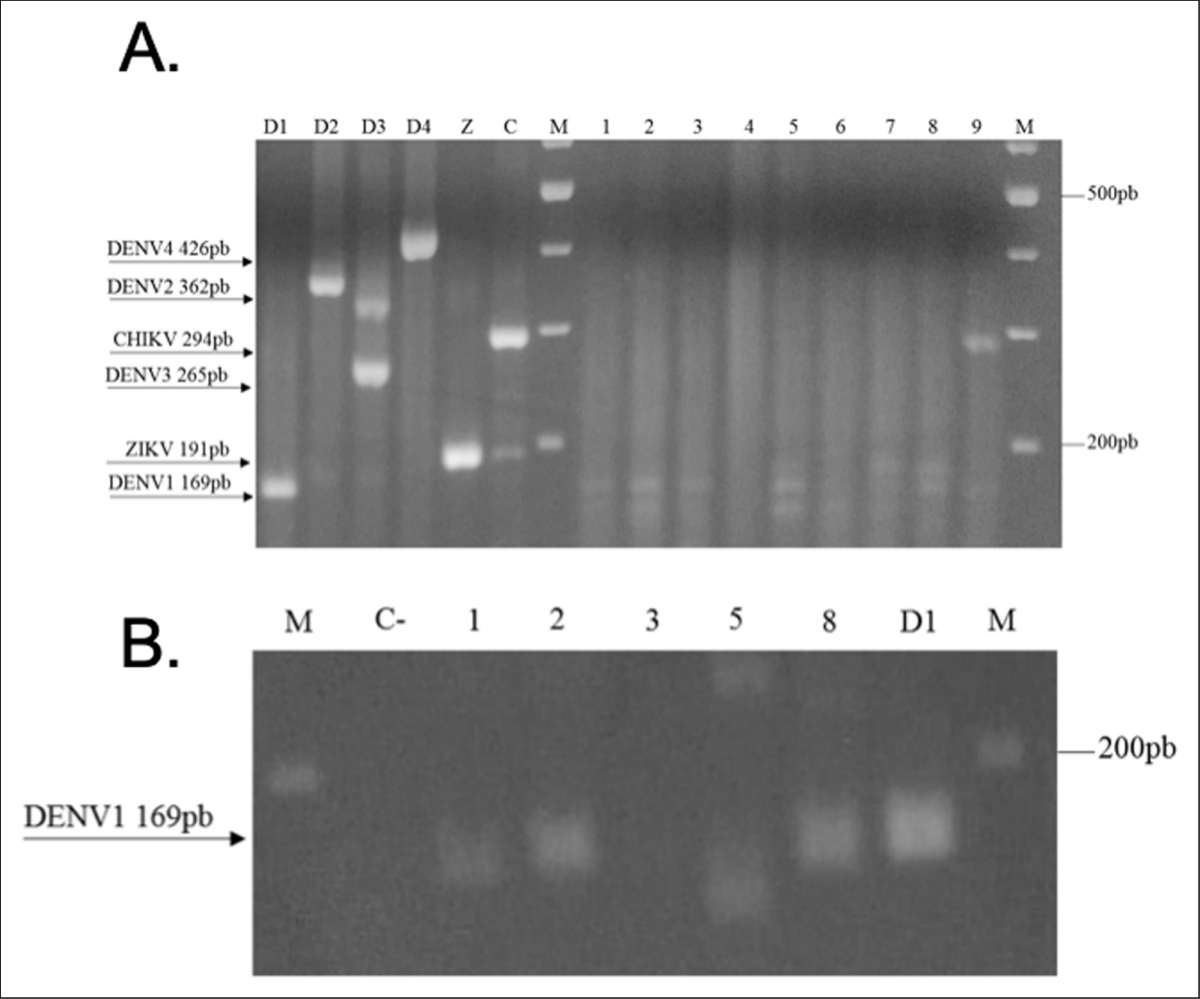
Identification of arboviruses in *A. aegypti* from Moniquirá. **A.** 2.5% agarose gel electrophoresis of the RT-PCR products visualized using the intercalating ethidium bromide. Positive controls D1: DENV-1 (169 bp), D2: DENV-2 (362 bp), D3: DENV-3 (265 bp), D4: DENV-4 (426 bp), Z: ZIKV (192 bp), C: CHIKV (294 bp). Line 1–9: Pools of A. aegypti. M: 100 bp molecular weight marker. **B.** Confirmation of infection with dengue virus DENV-1. Agarose gel electrophoresis at 2.5% of the species-specific RT-PCR products visualized with the intercalator ethidium bromide. C–: Negative control, 1–3, 5, and 8: pools of A. *aegypti*. D1: positive control DENV-1 (169 bp). M: 100 bp molecular weight marker.

A total of 68 *A. aegypti* adult mosquitoes were collected, of which 25 were females (36.76%) and 43 males (63.24%). Molecular analysis by RT-PCR showed infection with dengue virus serotype DENV-1 in three of the nine analyzed pools (Figure 3A). This result was confirmed with specific primers for this serotype (Figure 3B). Additionally, one of the nine pools were positive for the chikungunya virus (CHIKV). In total, 44.4% of the analyzed pools were positive for arboviruses (Figure 3A). Finally, of the three pools of females infected with dengue virus, one consisted of four individuals and the remaining two by three specimens. The pool infected with chikungunya virus consisted of three individuals. The MIR calculated for DENV-1 was 44.1 and for CHIKV 14.7.

## Discussion

Surveillance, prevention and control programs of arboviruses transmitted by *A. aegypti* (dengue, Zika, and chikungunya) in Colombia are based on the implementation of the integrated management strategy (EGI, in Spanish), where the use of insecticides is the most widely carried out strategy throughout the country to reduce vector populations [15,25]. However, the lack of infrastructure and specialized technical staff in endemic areas makes it difficult to monitor the state of susceptibility to insecticides, the mechanisms of resistance developed, and the construction of an early warning system from the virologic surveillance of the vector. These situations prevent timely decision-making and more rational use of insecticides. This work reports for the first time in Colombia, the simultaneous evaluation of susceptibility to insecticides (organophosphate and pyrethroid), the presence of mutations (*kdr*) related to insecticide resistance, and natural infection with arbovirus in *A. aegypti* from the municipality of Moniquirá, one of the cities with the highest transmission in the department of Boyacá.

The lack of knowledge in regions with high transmission of arboviruses by *A. aegypti* puts at risk the vector control programs that still depend on the use of these insecticides. The results showed tolerance to lambda-cyhalothrin pyrethroid in the “Moniquirá” strain. Similar results have been reported in other Colombian regions [8,9,10,16,26,27]. This generalized state of loss of susceptibility to this insecticide in the country has been mainly related to (i) metabolic resistance due to the increase in the activity of detoxification enzymes [8,9,16,27,28,29]; (ii) Cross-resistance with organophosphates by high selection pressure with temephos which has been widely and intensively used in the country [8,16,28], and (iii) presence of *kdr* mutations by pyrethroid selection pressure used both institutional level as community [10,11,12]. It is possible that the loss of susceptibility to lambda-cyhalothrin reported in this work, is related to the prolonged use, during ten years, of organophosphate and pyrethroids in the department of Boyacá for the control of *A. aegypti* and triatomines (Personal communication of health local secretary, Boyacá).

Nowadays, about eleven *kdr* mutations in the sodium channel of *A. aegypti* have been linked around the world with pyrethroid resistance [30]. In Colombia, mutations V1016I and F1558 were reported in populations with pyrethroid resistance phenotype in the Caribbean Region, Coffee region, Meta, and Antioquia [10,11,12]. Recently, the V419L mutation was associated with resistance to lambda-cyhalothrin in populations of Riohacha and Villavicencio [10]. The present work reports the presence of these three *kdr* mutations in a population of mosquitoes with tolerance to lambda-cyhalothrin, in the municipality of Moniquirá, department of Boyacá. It is noteworthy that frequencies of mutated alleles at positions 419L, 1016I, and 1558C of the sodium channel gene in the “Moniquirá” strain were 0.39, 0.4, and 0.95, respectively. On the other hand, the comparison of the observed and expected genotypic frequencies did not show the Hardy-Weinberg gene disequilibrium, indicating that selection pressures are not acting on these populations. There are few studies developed in the country, in which the presence of mutations and loss of susceptibility to type II pyrethroids are related. The results of this study support the results of Granada and collaborators 2018 since the loss of susceptibility in *A. aegypti* of the municipality of Moniquirá seems to be more related to a high frequency of the alleles 1016I and 419L, than with a high frequency of the mutated allele 1558C. The populations of Colombia analyzed by Granada et al., had a high frequency of the 1558C mutated allele, and this mutation has been implicated in resistance to other insecticides such as type I pyrethroids. Thus, it will be interesting to evaluate shortly whether the “Moniquirá” strain is resistant to permethrin and another type I pyrethroid.

On the other hand, the susceptibility test with the organophosphate malathion showed that the “Moniquirá” strain was susceptible to this insecticide. Similar results have been reported in the departments of Antioquia, Putumayo, Chocó, Nariño, Cauca, Atlántico, Casanare, and Caldas [8,16,26,27,29]. However, losses of susceptibility to this insecticide attributed to its intensive use have been reported in Colombia and other countries of the continent [8,31,32]. According to the health Secretary of Boyacá, malathion was used between 2007 and 2015 as measures of crashes in confirmed cases of dengue and severe dengue. Additionally, during the last two years, this adulticide has been replaced by deltamethrin, a practice that has been suggested as the most appropriate way to maintain susceptibility to date [8,16]. With the results of this study, it is advised to use the deltamethrin insecticide rationally and alternately, using appropriate doses to control *A. aegypti*. The insecticide deltamethrin belongs to the same group of lambda-cyhalothrin, evaluated in this work, a situation that may explain the high frequency of mutated alleles 419L and 1016I in this population.

Furthermore, the infection of the pools analyzed suggests that in the municipality of Moniquirá there was a higher dynamics of dengue virus transmission than those reported in other areas of Colombia during epidemic periods [33,34]. The levels of natural infection of 44.1% here detected are similar to those reported in Armero-Guayabal (33%), and superior to those of Valle del Cauca 12.7%, Bello 16.8%, Villavicencio 11,18%, Riohacha 9,62%, and Medellín 32,42% [33,34,35,36]. However, higher rates than those reported in this study were found for Cundinamarca (62%) during 2013 [37]. We consider that the levels of infection shown here are due to the characteristics of the transmission of dengue in the municipality of Moniquirá, and are not consequences of contamination during the diagnostic process, since all the pools were processed simultaneously, obtaining infections with different arboviruses and negative pools [37].

The transmission of dengue in the department of Boyacá is endemic-epidemic, with cyclical behavior and reports of outbreaks in its territory since 1992 [3]. During 2016 and 2017, this department presented an incidence of dengue cases per 100,000 inhabitants above the average recorded for these years in Colombia, suggesting a high intensity of transmission [5,6]. The high rate of natural infection reported for *A. aegypti* de Moniquirá draws attention because the year 2017 was not a year of an epidemic outbreak, as it did during 2016 [5]. However, the registered natural infection can be explained by a high circulation of the virus in humans without clinical symptoms, as has been reported elsewhere in Colombia and Brazil in periods after an epidemiological outbreak [34,38,39]. Additionally, we did not rule out that efficient vertical transmission of DENV-1 contributed to the infection levels obtained in *A. aegypti*, as has been demonstrated by other authors [40].

DENV-1 is one of the serotypes with the highest circulation and prevalence in Colombia, being detected in humans during all the epidemic periods registered in the country between 1971 and 2010 [3]. In the present study, this serotype was the only one found in *A. aegypti* in the study area. The serotype DENV-1 has been reported at an entomological level in different studies carried out in Colombia, infecting *A. aegypti* in Antioquia, Valle del Cauca, Tolima, and Cundinamarca [33,34,37]. Additionally, this serotype was the most prevalent in humans (77%) in the department of Boyacá during 2016 and 2017 of agreements with the departmental public health laboratory data (unpublished data).

To date, there are few reports of natural infection of *A. aegypti* with chikungunya in Latin America [41,42,43]. The present work contributes to the contribution of data in this sense since it presents evidence of natural infection of *A. aegypti* of 14.7% with chikungunya in Colombia. In the department of Boyacá, chikungunya does not display the intensity of transmission that dengue presents. However, the occurrence of cases of this arbovirosis has been recorded since 2014, and in 2017 the national surveillance system recorded the appearance of 12 cases for this department, one of these in the municipality of Moniquirá [6]. Although the positive pool was likely made up of non-infective females, it is also possible that at the time of diagnosis the virus would have completed its extrinsic cycle. Therefore, we suggest the adoption of virologic surveillance strategies for the study area and that, besides, it should include other arboviruses such as chikungunya and Zika.

In the present study, the baseline of the resistance state and the presence of *kdr* mutations in a natural population of *A. aegypti* from one of the areas with the highest transmission of dengue and other arboviruses within the department of Boyacá. The presence of *kdr* mutations and the loss of susceptibility to type II pyrethroids are sufficient for the adoption of measures that prevent the increase of the frequencies of pyrethroid resistance alleles type I and II. Thus, more controlled use of institutional insecticides, the rotation of these, and the use of alternatives to control *A. aegypti* that contemplate the mobilization and education of the community are necessary. These measures, accompanied by a monitoring of the circulation of arboviruses should be implemented in Moniquirá and other municipalities of the department to build a strategy tailored to the needs of this region of the country.

## Conclusions

The presence of *kdr* mutations and the loss of susceptibility to type II pyrethroids are sufficient for the adoption of measures that prevent the increase of the frequencies of pyrethroid resistance alleles type I and II. Thus, more controlled use of institutional insecticides, the rotation of these, and the use of alternatives to control *A. aegypti* that contemplate the mobilization and education of the community are necessary. These measures, accompanied by a monitoring of the circulation of arboviruses should be implemented in Moniquirá and other municipalities of the department to build a strategy tailored to the needs of this region of the country.
